# Interconnect for Dense Electronically Scanned Antenna Array Using High-Speed Vertical Connector

**DOI:** 10.3390/s23208596

**Published:** 2023-10-20

**Authors:** Nooshin Valizade Shahmirzadi, Natalia K. Nikolova, Chih-Hung Chen

**Affiliations:** Department of Electrical and Computer Engineering, McMaster University, Hamilton, ON L8S 4L8, Canada; talia@mcmaster.ca (N.K.N.); chench@mcmaster.ca (C.-H.C.)

**Keywords:** antenna arrays, large-scale antenna arrays, antenna array feeds, high-speed vertical connector, microwave interconnects, microwave imaging, ultra-wideband frequency range

## Abstract

We present the design and the performance evaluation of a new interconnect for large-scale densely packed electronically scanned antenna arrays that utilize a high-speed digital board-to-board vertical connector. The application targets microwave tissue, imaging in the frequency range from 3 GHz to 8 GHz. The tissue-imaging arrays consist of hundreds of active antenna elements, which require low-reflection, low-loss, and low-crosstalk connections to their respective receiving and transmitting circuits. The small antenna size and the high array density preclude the use of coaxial connectors, which are also expensive and mechanically unreliable. Modern board-to-board high-speed connectors promise bandwidths as high as 12 GHz, along with high pin density, mechanical robustness, and low cost. However, their compatibility with the various transmission lines leading to/from the miniature printed antenna elements and microwave circuitry is not well studied. Here, we focus on the design of the transitions from coplanar waveguide transmission lines to/from a high-speed vertical connector. The performance of the interconnect is examined through electromagnetic simulations and measurements. Comparison is carried out with the expensive sub-miniature push-on sub-micro coaxial connectors commonly used in miniature radio-frequency electronics. The results demonstrate that high-speed vertical connectors can provide comparable performance in the UWB frequency range.

## 1. Introduction

Over the past decade, the development of electronically scanned antenna arrays (ESAAs) has been the focus of intense research since they outperform mechanically scanned systems with exceptional speed, compactness, lightweight, and immunity to positioning errors. These advantages make the ESAAs the preferred choice in applications such as wireless communications [1,2,3,4], radar systems [5,6], nondestructive testing [7,8], and microwave imaging [9,10,11,12], among others. In communications, these arrays enable dynamic beamforming, where the antenna pattern is shaped to maximize the signal power toward the desired receiver(s) and/or to minimize interference from other directions. Radar systems utilize ESAAs for precise beam steering to acquire data from different angles and positions, resulting in improved sensitivity, image quality, and resolution. In nondestructive testing and imaging, ESAAs primarily offer a multiplexing capability among transmit/receive channels, where the inputs/outputs of multiple antenna elements are combined to connect to a single receiver or transmitter.

To realize ESAAs in the microwave frequency range, a widely adopted approach integrates solid-state phase shifters [4,13,14], radiofrequency (RF) switches [9,15,16], p-i-n and Schottky diodes [17,18,19,20], varactors [21,22,23], etc., within each antenna element. Such integration adds functionality to the sensing element (e.g., frequency conversion, tunable phase delay), but it does not resolve the problems arising when multiplexing hundreds of such sensors to the transmitting/receiving circuitry as well as the digital control circuitry. In [21], a complementary circuit composed of power dividers and varactors is developed at the rear of the array’s printed circuit board (PCB) to provide beam-steering capability at 3 GHz. The topology is narrowband and limited to controlling only 16 elements. Substantial modifications would be necessary to scale up the number of elements to several hundred and to overcome the bandwidth limitations of the power dividers. In a recent study [4], eight solid-state phase shifters are connected to 32 ports of a phased array antenna by utilizing a combination of one 1-to-8 power splitter and eight 1-to-4 power dividers, all interconnected through RF cables. This configuration ensures highly uniform amplitudes and accurate phase shifts. However, the design is bulky. In ESAAs currently employed in microwave tissue imaging [9,10,11,12], the transmitting/receiving circuitry includes a network of RF switches which are connected through high-quality RF cables and coaxial connectors to the antenna elements. Due to losses in the RF switches and the large size of the coaxial connectors, there are limitations on the number of multiplexed elements and the inter-element spacing. Thus, complementary mechanical scanning is usually required to increase the spatial sampling density [10,12].

Efficient RF signal routing to/from the mm-sized antenna elements within densely packed arrays is a significant challenge, particularly in the context of imaging, where high-density ESAAs must provide sufficient spatial sampling without the need for mechanical scanning. Conventional RF switching networks are not practical when multiplexing hundreds of array elements. This is due to their substantial insertion loss, limited isolation, and calibration difficulties. To overcome these limitations, a solution based on multiplexing at a single-tone intermediate frequency (IF) has been proposed in [24,25]. In a recent study on microwave imaging of breast tissue [25], a planar ultra-wideband (UWB) active receiving array has been introduced in the frequency range of 3 GHz to 8 GHz. This array design allows for scalability to a larger configuration of 16 × 16 elements, where an IF switching network enables the multiplexing of hundreds of sensors within the array.

**Figure 1:**
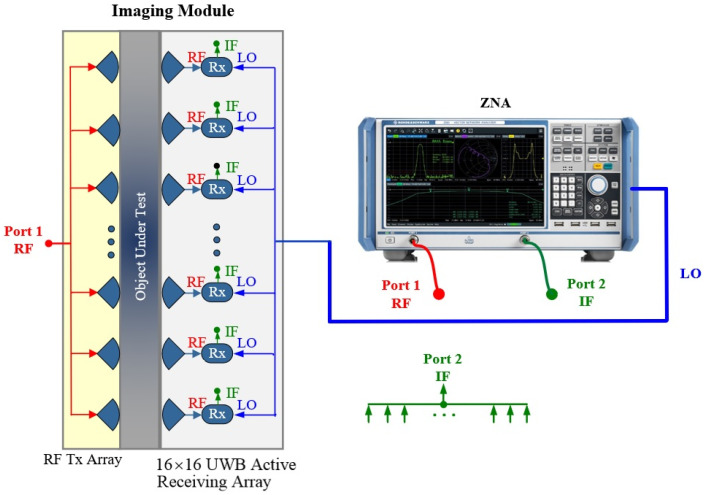
The architecture of an electronically scanned system based on the proposed UWB active receiving array prototype in [25] for breast-tissue microwave imaging.

Figure 1 shows the architecture of a UWB electronically scanned microwave compressed-breast imager employing a scaled-up prototype of the active receiving array first proposed in [25]. The architecture is composed of a 2-port vector network analyzer (VNA), an RF transmitting (Tx) array fed by the VNA through a power distribution network, a local oscillator (LO) power distribution network, a UWB active receiving (Rx) array that captures the scattered signal through an object under test, and an IF-switching network. The employed VNA [26] features an additional internal signal generator, which provides an RF-synchronized LO signal for down-conversion. This allows for vector (magnitude and phase) frequency-conversion measurements. The passive antenna elements in the Tx and Rx arrays are the same. The UWB active Rx array in this development is composed of 16 × 16 sensors, where each antenna element is equipped with its own RF front end down-converting the UWB RF signals to a single-tone IF signal. The spacing between the elements is 12 mm in both lateral directions. Due to space constraints and the lack of a single-chip UWB platform capable of amplifying and down-converting RF signals, each array element is integrated only with a low-noise amplifier (LNA) chip [25]. The mixer arrays, the LO distribution network, and the IF-switching network reside on a separate board, necessitating a low-reflection, low-loss, and low-crosstalk board-to-board transition.

This work presents a solution that achieves seamless transitions from the UWB 16 × 16 active Rx array PCB to a separate PCB of the corresponding array of mixers and the IF switching network which multiplexes the 256 sensors at the single-tone 30 MHz IF output. To this end, we explore the use of high-speed vertical connectors, widely employed in computer technology. These connectors offer several advantages over conventional coaxial connectors, including high pin density, low cost, and mechanical robustness, making them a much-preferred choice for board-to-board transitions. The primary focus of this study is to design a high-quality RF transition between the grounded coplanar waveguide (GCPW) lines at the outputs of the LNAs on the Rx antenna board (described in [25]), and a high-speed vertical connector. We assess the performance of this interconnect through electromagnetic (EM) simulations and measurements of fabricated test boards. Subsequently, the high-speed vertical connectors are incorporated into the 16 × 16 UWB active receiving array, and their performance is investigated and compared with sub-miniature push-on sub-micro (SMPS) coaxial connectors in a 6 × 6 UWB active Rx array prototype [25]. The SMPS connectors, along with their respective cables and adapters, are expensive. A single SMPS connector costs over US $20, which leads to substantial expenses when arrays consisting of several hundred elements are to be interfaced with electronics. They are mechanically fragile with a limited number of mating cycles, usually ranging from 100 to 500, depending on whether they employ a full detent or smooth bore interface. Importantly, connecting, and disconnecting hundreds of such connectors, densely packed on a PCB, is difficult, leading to unreliable electrical performance. Hence, the SMPS connectors are deemed unsuitable for connectorizing large arrays.

To the best of our knowledge, no existing antenna array has yet harnessed the potential benefits of a high-speed connector in the UWB frequency range for board-to-board connections. Therefore, our proposed vertical board-to-board transition holds significant promise not only for our specific application but also for other scenarios that require a transfer of microwave signals between high-density PCBs with hundreds of input/output GCPW interconnects.

In Section 2, the design of the GCPW interconnects for high-speed vertical connectors is discussed, followed by validation through measurements. Section 3 focuses on the implementation of the designed GCPW interconnects on the 16 × 16 UWB Rx array board together with the vertical board-to-board connector. The RF performance of the assembly is validated through measurements and compared with that of the SMPS connectors terminating the elements of a prior 6 × 6 UWB array prototype [25]. Discussion and conclusions are presented in Section 4 and Section 5, respectively.

## 2. Vertical Board-to-Board Interconnect Design

In [25], a planar receiving array of UWB active slot antennas has been proposed for microwave imaging of the compressed breast with operating bandwidth from 3 GHz to 8 GHz. Each array element integrates an LNA chip to boost the received signal. This is necessary in tissue imaging because microwave signals suffer significant attenuation, which grows rapidly with frequency [27,28,29]. Using the published permittivity and conductivity values for the various healthy breast tissue properties [27], along with a simple plane-wave propagation model, it is possible to estimate the attenuation for a signal path of about 60 mm (the average thickness of a compressed breast during the microwave measurement). Depending on the breast tissue composition in terms of fibro-glandular and adipose content, the estimated attenuation is between −60 dB and −90 dB at the highest frequency of 8 GHz. This translates into attenuation rates from −10 dB/cm to −15 dB/cm. These estimates are confirmed in [25], where a transmission measurement shows an attenuation of about −42 dB at 8 GHz through a 33 mm-thick breast-tissue phantom, which mimics a BI-RADS (Breast Imaging Reporting and Data System) Category B healthy breast tissue (scattered areas of fibro-glandular tissue with 25% to 50% of the overall breast-tissue mass [30]). Thus, signal amplification on receive is imperative, and the active array in [25] employs LNA chips with a flat 20-dB gain throughout the 3 GHz to 8 GHz bandwidth.

**Figure 2:**
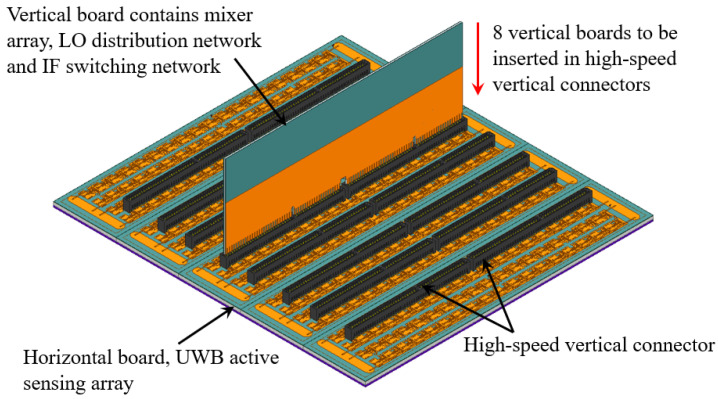
Isometric view of a 16 × 16 UWB active sensing array with 8 rows of vertical connectors, where each row consists of two edge-to-edge MEC2-50-01-L-DV connectors. One of the eight vertical boards (carrying additional electronic circuits) before insertion into the connector is also shown.

The initial 6 × 6 array prototype reported in [25] demonstrates a seamless integration of the LNA chips with the slot antennas on the Rx array PCB. The design is scalable, which allows for its expansion into larger arrays, such as the 16 × 16 Rx array shown in Figure 2. However, the initial 6 × 6 array prototype uses SMPS coaxial connectors, which are impractical for large arrays, as discussed earlier. We reiterate that the system architecture in Figure 1 requires each Rx array element to connect to a dedicated mixer, requiring 256 identical high-quality RF interconnect paths.

To address this problem, we propose to employ off-the-shelf high-speed board-to-board vertical connectors that offer high pin density, mechanical robustness, and a low cost (below US $10 per connector). Figure 2 shows the horizontal PCB of the large UWB active Rx array along with the mounted connectors (Samtec MEC2-50-01-L-DV [31]). The connector features 50 pins with a 2-mm pitch on each side. Since the center-to-center spacing between the elements of the Rx array is 12 mm, the connector accommodates 8 elements on each side (16 array elements in total). As shown in Figure 2, two connectors, mounted edge-to-edge, can accommodate two 16-element rows (32 array elements in total). Overall, 16 vertical connectors are required for the assembly. The connectors link the Rx array PCB to eight identical vertical PCBs, each accommodating 32 mixer chips, along with an LO distribution network and an IF switching network. Note that the PCB of the Rx antenna array in Figure 2 contains unconnected elements at its periphery. These are dummy elements, which ensure identical performance of the connected array elements, all of which are at least one array element away from the PCB edges. In [25], the outputs of the LNAs on the active sensing array employ short GCPW transmission lines (TLs) terminated with SMPS connectors. As discussed later, these GCPWs need to be thoroughly redesigned in order to achieve acceptable reflection and transmission coefficients when interfacing them with the pins of the vertical board-to-board connector. Specifically, the design goal is to achieve reflection coefficients below −10 dB and “through” transmission coefficients above −1 dB in the frequency band from 3 GHz to 8 GHz.

### 2.1. EM Design of the GCPW Interconnect for the High-Speed Vertical Connector

The documentation on MEC2-50-01-L-DV [32] suggests a 50-Ω microstrip layout for the connector’s pin footprint. However, in the UWB frequency range, GCPWs are preferred over microstrip lines due to lower dispersion, lower loss, and lower channel crosstalk. Therefore, a new design is necessary for GCPW leads between the circuitry and the connector. To this end, the HFSS [33] encrypted model of MEC2-50-01-L-DV has been acquired from the manufacturer to perform the design using full-wave EM simulations.

**Figure 3:**
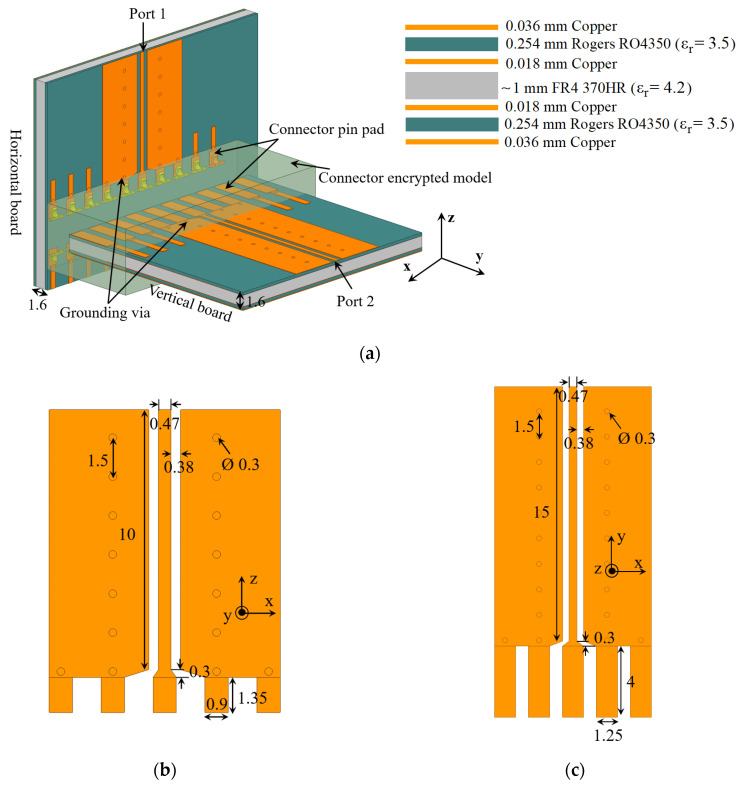
(**a**) Isometric view of the assembly consisting of the GCPW TLs test boards and the encrypted model of the high-speed vertical connector, alongside the stack-up of the test boards. Circles in grey indicate grounding via holes. For a better view of the pin footprint of the vertical connector, the assembly is tilted at an angle such that the horizontal (Rx antenna array) board appears vertical in the CAD drawing, whereas the vertical (mixer array) board appears horizontal. Layouts of the GCPWs leading to the vertical connector on: (**b**) the horizontal board and (**c**) the vertical board. All dimensions are in mm.

The initial design is based on the assumption that the connector might function effectively with a direct connection to 50-Ω GCPW transmission lines. Two test PCBs (horizontal and vertical) are designed to evaluate its performance with such GCPWs over the frequency range from 3 GHz to 8 GHz. Figure 3 shows the designed GCPW test boards, along with their respective layout and stack-up. Note that the connector mates to vertical boards of 1.6 mm thickness, which necessitates the FR4 layer as a filler in the middle of the stack-up shown in Figure 3a. The signal trace width and the gap width of the GCPW are the same on both the horizontal and vertical boards. The trace width is 0.47 mm, and the gap width is 0.38 mm. Grounding via holes with 1.5 mm center-to-center spacing is employed in the ground planes of GCPWs. 

Wave-port analysis of the GCPW traces in HFSS yields an approximate 50-Ω characteristic impedance averaged over the 3 GHz to 8 GHz bandwidth. The pin pads’ dimensions for both the horizontal and the vertical boards are dictated by the HFSS encrypted model of the connector. The HFSS encrypted model of the connector contains only 20 pins, 5 of which are specifically designated for testing the GCPW interconnect. To prevent ground loops, all the remaining pins on both the vertical and horizontal boards are grounded. The reflection coefficient and transmission coefficient of this configuration are assessed using a 2-port *S*-parameter analysis, employing wave-port excitation in HFSS with a frequency sweep from 3 GHz to 8 GHz. 

**Figure 4:**
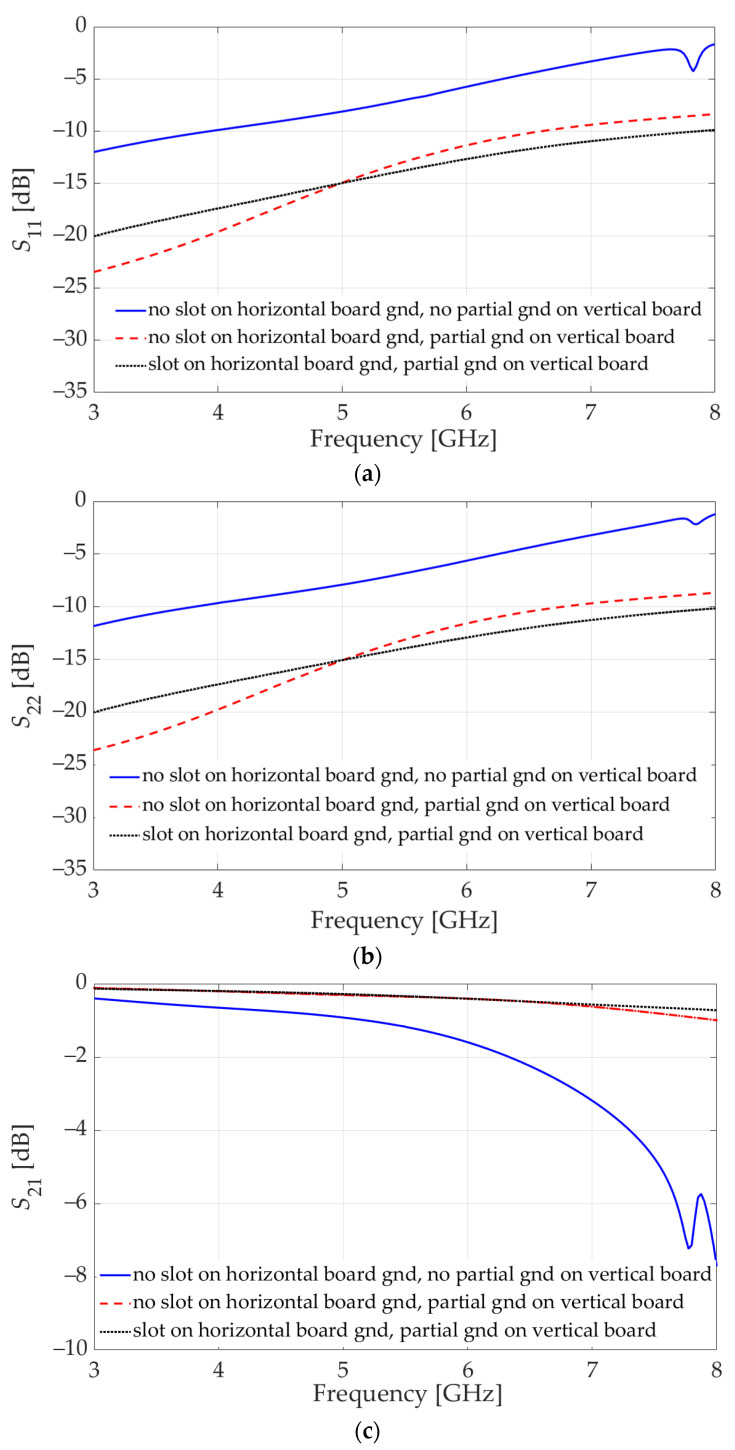
Simulation results for the initial and improved designs of the GCPW layouts on the horizontal and vertical boards in the vertical connector assembly. The improvement is due to two major design changes: (i) introducing partial ground planes on the vertical board and (ii) introducing a slot in the horizontal board ground layer. The initial design results are shown with solid blue lines. The major improvement due to design change (i) is shown by the dash-dot red line. The additional improvement due to design change (ii) is shown by the dotted black line. (**a**) Reflection coefficient at Port 1 (*S*_11_). (**b**) Reflection coefficient at Port 2 (*S*_22_). (**c**) Transmission coefficient (*S*_21_).

The simulation of this initial design indicates that the reflection coefficients at both ports (*S*_11_ and *S*_22_), surpass the −10 dB threshold beyond 4 GHz; see the solid-line results in Figure 4a,b. Accordingly, the transmission coefficients (*S*_21_ = *S*_12_) are not satisfactory either, with values as low as −7 dB at high frequencies; see Figure 4c. We note that *S*_11_ and *S*_22_ are not identical since the vertical connector does not have a mid-point symmetry.

**Figure 5:**
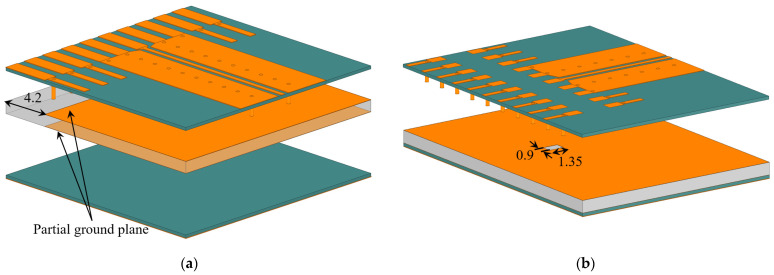
Design improvements at the contact points between the GCPWs and the vertical connector: (**a**) partial ground planes on the vertical board, where metallization is partially removed from the sections to be inserted into the connector; (**b**) the slot in the ground plane of the horizontal board. Circles in grey and cylinders indicate grounding via holes.

Since the HFSS model of the connector is encrypted, its internal composition is inaccessible, preventing the analysis of the causes for the unsatisfactory impedance-match performance. However, the input resistance and reactance can be inspected at each port of the configuration in Figure 3a. At higher frequencies, especially above 5 GHz, the input impedances at both ports exhibit parasitic reactances. To bring the reflection coefficients below the desired −10 dB level throughout the UWB frequency spectrum, two significant design changes are made. First, partial ground planes on both sides of the vertical board are introduced (see Figure 5a) such that the metallization is partially removed from the sections that are inserted into the connector. Second, a slot beneath the connector’s pin pad at the signal trace of the GCPW on the horizontal board is introduced, as shown in Figure 5b. Each one of these measures counteracts the parasitic effects at higher frequencies, as is asserted by the reflection and transmission coefficient plots in Figure 4. The improved design features a good impedance match (reflection loss better than 10 dB) in the whole band from 3 GHz to 8 GHz, except for a minor violation at 8 GHz. The corresponding improvement in the transmission coefficient is also significant so that it does not exceed 1 dB over the entire frequency range.

### 2.2. Measurement Validation of GCPW Interconnect for High-Speed Vertical Connector

**Figure 6:**
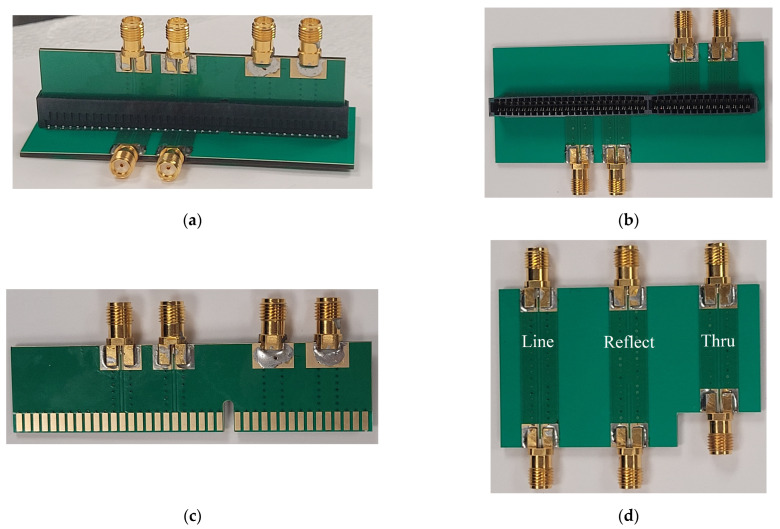
(**a**) Assembly showing the vertical test board inserted in the connector mounted on the horizontal test board. (**b**) GCPW horizontal test board with the mounted vertical board-to-board connector. (**c**) GCPW vertical test board. (**d**) GCPW calibration board with Thru, Reflect (Shorted GCPW), and Line GCPW TLs.

To verify the performance of the GCPW interconnects for the high-speed vertical connector, the test-board designs described in Section 2.1 are fabricated along with Thru, Reflect, and Line calibration boards (see Figure 6). These boards are equipped with edge SMA connectors for 2-port measurements with a vector network analyzer (VNA) (E8363B, Keysight Technologies). In the measurements, the MEC2-40-01-L-DV connector is employed in lieu of MEC2-50-01-L-DV, featuring a 40-pin configuration instead of 50 pins. Aside from the pin number, the 40-pin and 50-pin vertical connectors are identical, and their electromagnetic performance is also identical. Thus, the HFSS encrypted model of the MEC2-DV series vertical connector is valid for any pin count. The 8-term error model [34] is implemented in MATLAB [35] and utilized to de-embed the effect of the edge SMA connectors and their transitions to the GCPW TLs. The de-embedding requires the 2-port *S*-parameter measurements of the calibration 2-ports (Thru, Reflect, Line) shown in Figure 6d. 

**Figure 7:**
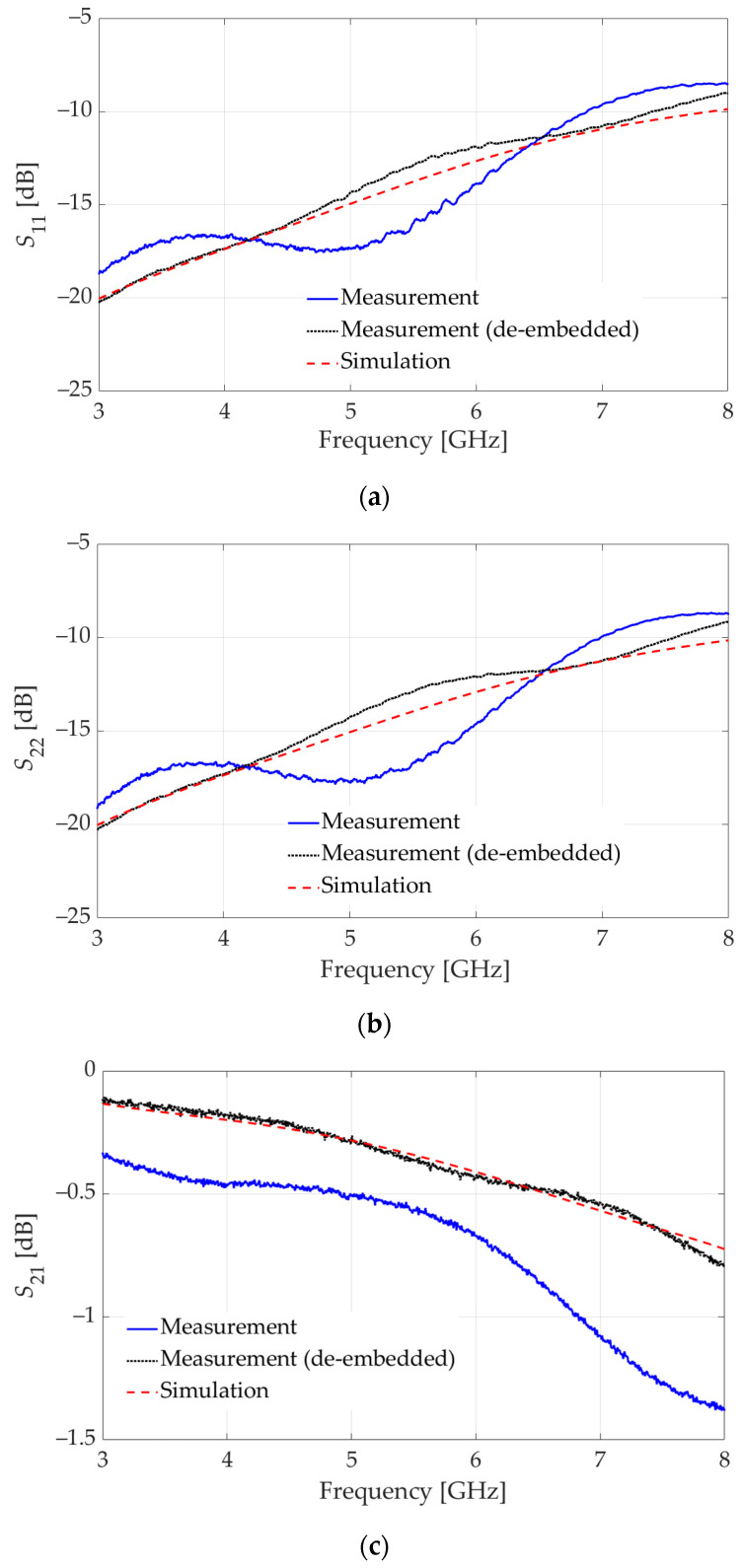
Measured, de-embedded, and simulated *S*-parameters of the vertical connector assembly with the GCPW test boards, both of which employ GCPW signal trace width of 0.47 mm and gap width of 0.38 mm: (**a**) reflection coefficient at Port 1, (**b**) reflection coefficient at Port 2; (**c**) transmission coefficient.

The *S*-parameters of the assembly in Figure 6a are also measured. The obtained four sets of *S* parameters (3 calibration measurements and one device measurement) are imported into the MATLAB code to extract the *S*-parameters of the high-speed vertical connector together with the pin transitions to GCPW TLs. The raw measured *S*-parameters of the assembly in Figure 6a are presented in Figure 7 along with the de-embedded *S*-parameters. It is observed that, despite the satisfactory simulation results in Figure 4, in measurements, the reflection coefficients at Port 1 and Port 2 violate the −10 dB threshold at higher frequencies.

**Figure 8:**
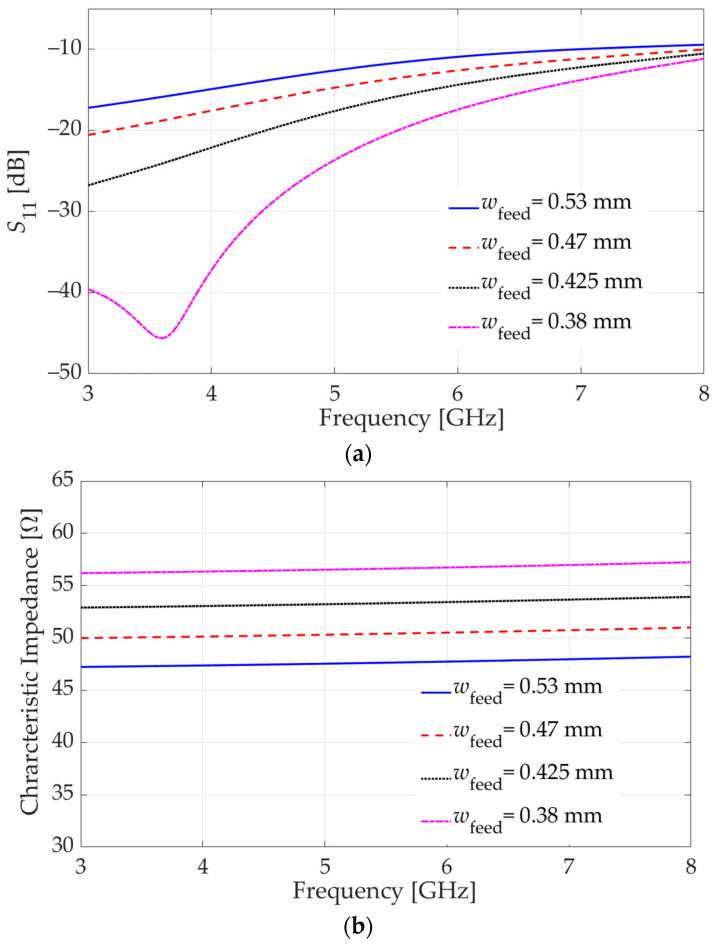
Effect of variation in the GCPW signal trace width (*w*_feed_) on: (**a**) the reflection coefficient at Port 1 (generalized *S*_11_), (**b**) the GCPW characteristic impedance on the horizontal board (Port 1). The gap width is fixed at 0.38 mm.

To address this problem, a parametric sweep is conducted in HFSS, varying the width of the GCPW signal trace while keeping the gap width intact (0.38 mm). Again, a wave-port excitation is employed. Representative results for the parametric sweep are shown in Figure 8a, which shows that a signal trace width of 0.38 mm along with a gap width of 0.38 mm provides a better impedance match with some margin below the −10 dB threshold. With this trace width, the HFSS wave-port analysis shows a characteristic impedance of 56 Ω on average over the frequency band from 3 GHz to 8 GHz, as shown in Figure 8b (*w*_feed_ = 0.38 mm). The reflection coefficient at Port 2 has similar behavior. 

**Figure 9:**
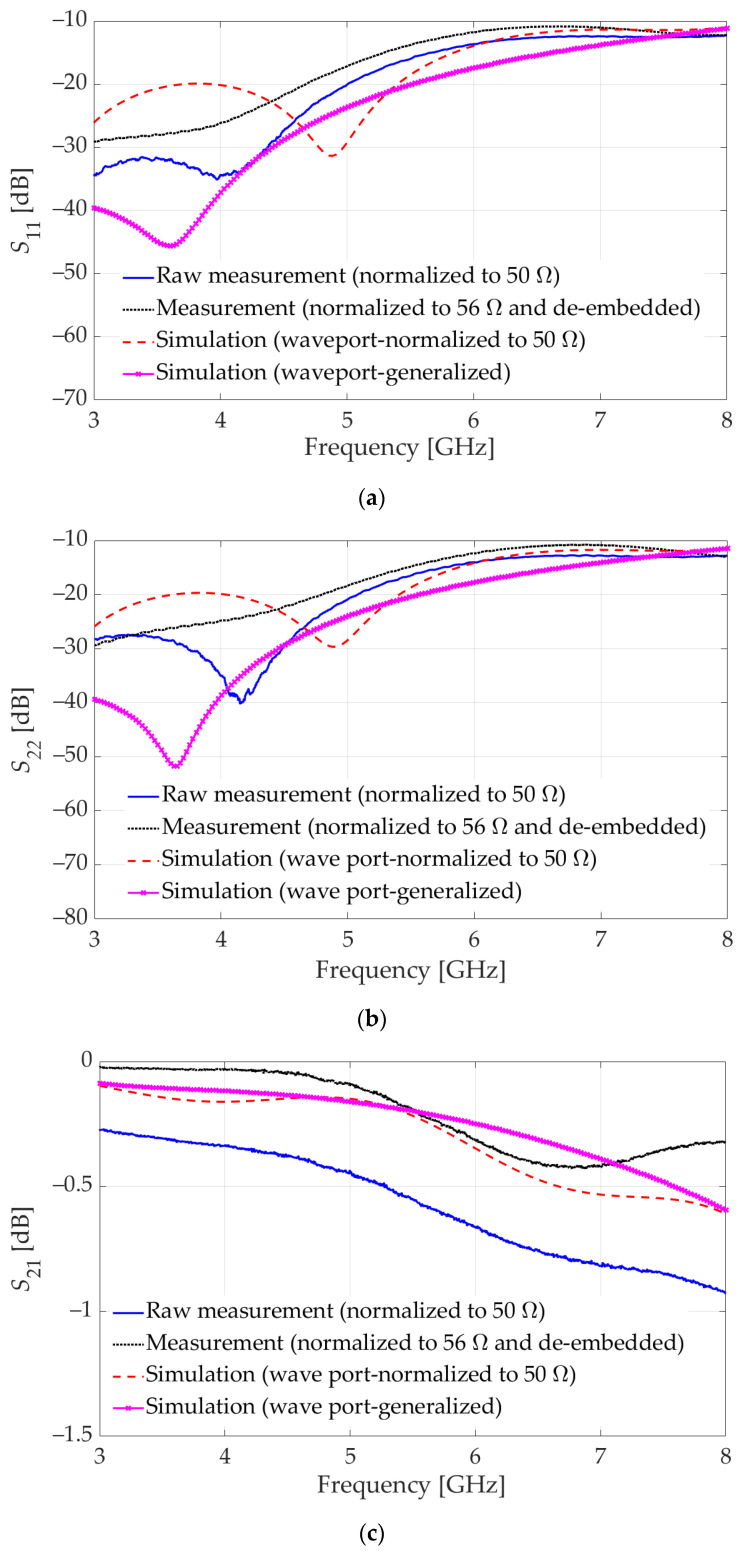
Measured (raw and de-embedded) and simulated *S*-parameters of the vertical connector assembly with test boards with GCPW signal trace width of 0.38 mm and gap width of 0.38 mm: (**a**) reflection coefficient at Port 1, (**b**) reflection coefficient at Port 2, (**c**) transmission coefficient.

Another set of GCPW calibration boards and test boards with this trace width *w*_feed_ = 0.38 mm have been fabricated and measured. Since the characteristic impedance of the GCPW TLs in these boards is 56 Ω on average, and the VNA reference impedance is 50 Ω, a reference impedance conversion [36] is carried out for all sets of measured *S*-parameters (the 3 calibration-board measurements and the assembly measurement) before the de-embedding with the 8-error-term model. Figure 9 shows the raw measured *S*-parameters of the assembly with the new test boards compared with simulation results. Figure 9 also includes the de-embedded *S*-parameters. Contrary to the first prototype, the measured and de-embedded reflection coefficients of the assembly with the new test boards are well below −10 dB, indicating that the high-speed vertical connector is better matched to 56-Ω GCPW interconnects. We note that there are some discrepancies between the simulated (generalized *S*-parameter) and measured *S*-parameters. The simulations employ wave-port excitations, where generalized *S*-parameters are computed (not normalized to a fixed system impedance). These *S*-parameters are expected to match better the de-embedded measured *S*-parameters, where the impact of the SMA connectors is removed and the reference system impedance is set to 56 Ω. This is indeed the case in Figure 9, especially at higher frequencies. At lower frequencies, the discrepancy appears larger; however, this is where the reflection coefficients are weak (below −20 dB), and the imperfections in the TRL calibration led to larger uncertainty on the dB scale. 

## 3. Integration of Vertical Connector with the UWB Active Sensing Array

The high-speed vertical connector is employed in the UWB active sensing array for microwave imaging applications. In such applications, it is important to achieve not only good impedance match but also good decoupling between receiving channels. This is investigated here utilizing a setup in which the connector is integrated with the actual 16 × 16 UWB active sensing array.

### 3.1. EM Simulation

**Figure 10:**
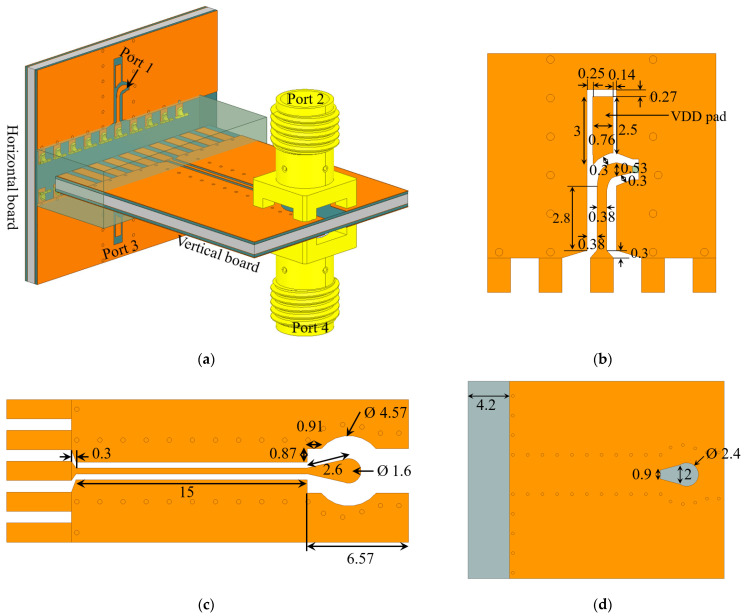
(**a**) Assembly showing the horizontal board with its transition path from the LNA to the vertical connector and the vertical board equipped with two SMA connectors for RF testing. (**b**) The top-layer layout on the horizontal board (LNA side) with dimensions in mm. The ground plane of this GCPW interconnect is the same as the one in Figure 5b. (**c**) The top-layer layout on the vertical board (SMA side) with dimensions in mm. (**d**) Partial ground plane of the vertical board with dimensions in mm. Circles in grey indicate grounding via holes.

Initially, the layout of the GCPW interconnect introduced in the preceding section is implemented on the horizontal board at the output of the LNA chip, which is represented simply with a 50-Ω lumped port in HFSS, as indicated in Figure 10a (Ports 1 and 3). The layout of this interconnect is shown in detail in Figure 10b. Note that the 56-Ω GCPW interconnect leading to the vertical connector is preceded by a carefully designed bend and a transition to a 50-Ω GCPW at the LNA output. These are critical for maintaining good impedance matches at Ports 1 and 3. The respective ground-plane layout is the same as the one in Figure 5b. Note that the vertical connectors accommodate two rows of antennas (16 on each side); thus, they must reside between the two antenna rows. This necessitates the right-angle bend from the LNA outputs toward the connector.

Also, the vertical board GCPW interconnect has been implemented on both sides of the board and has been equipped with SMA connectors for RF testing; see Port 2 and Port 4 in Figure 10a. The actual model of these SMA connectors is used in HFSS and they are excited by 50-Ω wave port excitations. The details of the GCPW layout at the top and bottom of the vertical board are shown in Figure 10c. The ground-plane layout for the two GCPWs on the vertical board is shown in Figure 10d. The purposeful creation of voids within the ground planes beneath the SMA connectors is critical for achieving good impedance matches on Ports 2 and 4. Another design feature is the transition from the GCPW to the footprint of the SMA connector, which impacts the impedance match as well. We reiterate the importance of the partial ground planes on the vertical board such that the metallization is removed in the PCB section that is inserted in the connector. These sections are visible in Figure 10d to the left of the partial ground plane.

**Figure 11:**
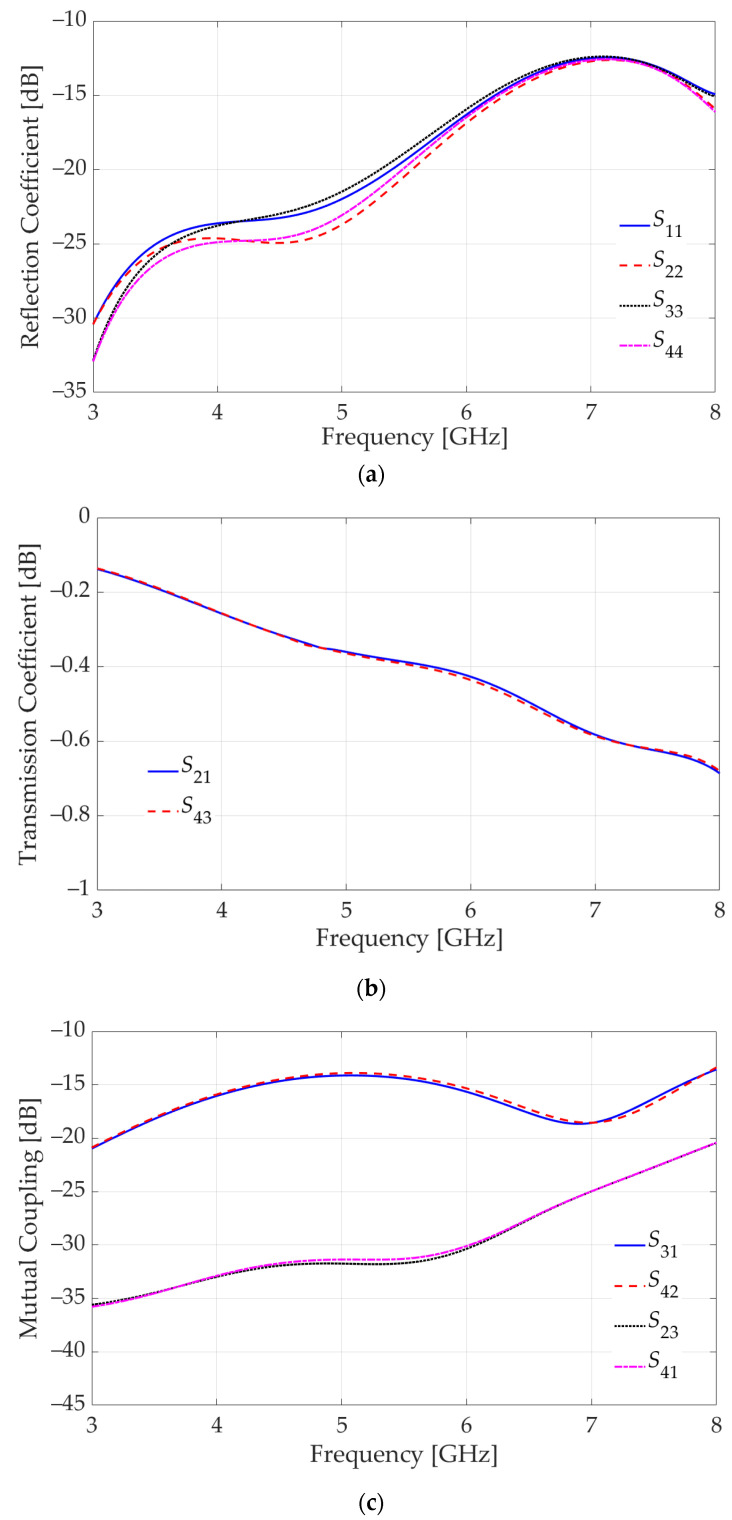
Simulated *S*-parameters of the configuration in Figure 10: (**a**) reflection coefficients in dB at all 4 ports, (**b**) “through” transmission coefficients between Ports 1 and 2 as well as Ports 3 and 4, (**c**) “mutual-coupling” (crosstalk) transmission coefficients.

A 4-port *S*-parameter analysis with a frequency sweep from 3 GHz to 8 GHz is performed in HFSS. The port assignment is indicated in Figure 10a. The 4-port *S*-parameters are shown in Figure 11. As observed in Figure 11a, the reflection coefficients are well below −10 dB. The “through” transmission coefficients between Ports 1 and 2, as well as Ports 3 and 4, are plotted in Figure 11b. They are better than −1 dB, which indicates high-quality transmission.

Figure 11c summarizes the mutual-coupling transmission coefficients, which describe undesirable crosstalk between ports. In imaging, it is important to channel the signal received by a sensor to its respective output but not to outputs associated with its neighbors. Thus, Port 1 should “talk” to Port 2, but not to Port 4. Similarly, Port 3 should “talk” to Port 4, but not to Port 2. Therefore, the crosstalk between Ports 1 and 4 as well as Ports 2 and 3 must be below −20 dB, and the respective *S*-parameters (*S*_41_ and *S*_23_) meet this goal.

**Figure 12:**
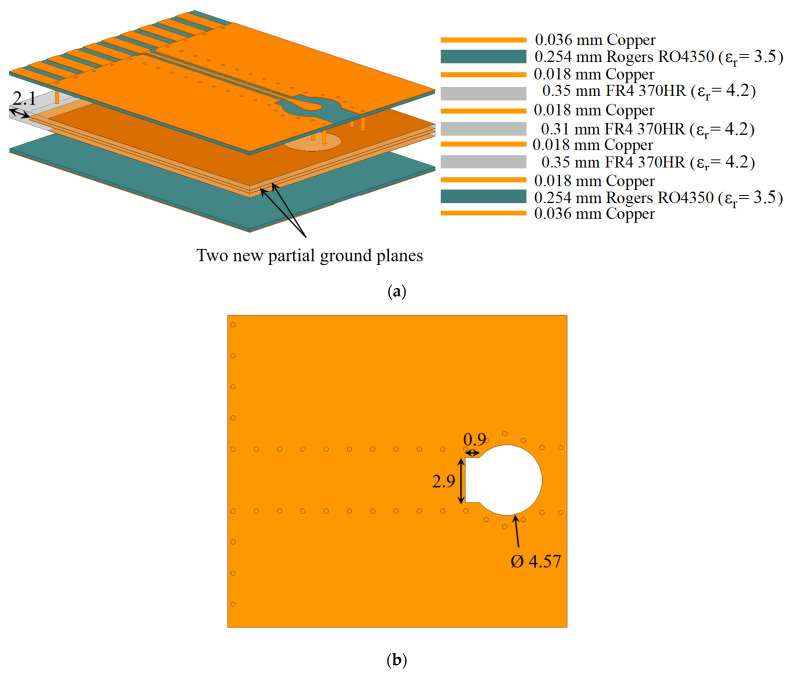
(**a**) Placement of two additional partial ground planes on the vertical board. (**b**) Adjustments were made to the slots in the ground planes directly beneath the SMA connectors. Circles in grey and cylinders indicate grounding via holes.

On the other hand, the crosstalk between Ports 2 and 4 (both on the vertical PCB) is less important in our application since both of these ports are outputs, i.e., there is no signal injection at these ports. These are ports leading to the RF inputs of separate mixers. Similarly, the crosstalk between Ports 1 and 3 (both on the horizontal PCB) is less important since a crosstalk due to a signal from the LNA at one of these ports is dissipated by the output resistance of the LNA at the other port. However, in other applications, it may be important to suppress this crosstalk represented by the transmission coefficients *S*_31_ and *S*_42_. As is evident from Figure 11c, these *S*-parameters exceed the threshold of −20 dB significantly (by more than 5 dB). To mitigate this crosstalk, two additional partial ground planes (one ground plane per GCPW) are introduced on the vertical board as shown in Figure 12a. A parametric sweep is executed on the length of the de-metallized segment, leading to the optimal choice of 2.1 mm as indicated in Figure 12a. However, the inclusion of these additional ground planes negatively impacts all reflection coefficients. To counteract this effect, the ground-plane slots beneath the SMA connectors are modified as shown in Figure 12b. 

**Figure 13:**
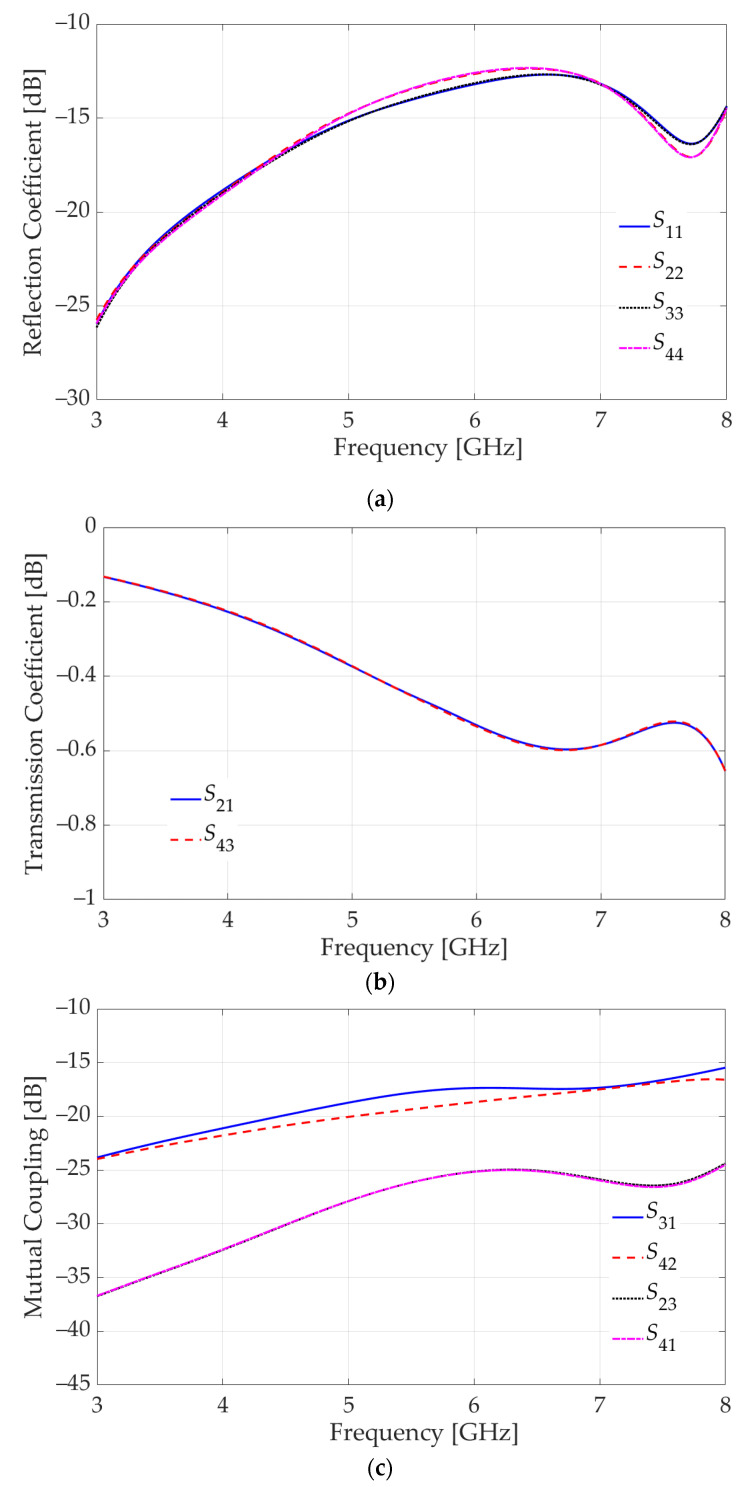
Simulated *S* parameters of the modified configuration presented in Figure 12: (**a**) reflection coefficients in dB at 4 ports, (**b**) “through” transmission coefficient between Ports 1 and 2 as well as Ports 3 and 4, (**c**) “mutual-coupling” (crosstalk) transmission coefficient between ports.

The *S*-parameters of the final design are plotted in Figure 13. The reflection coefficients (Figure 13a) remain below −10 dB, and the “through” transmission coefficients (Figure 13b) are better than −1 dB. The crosstalk (Figure 13c) between Ports 2 and 3, as well as Ports 1 and 4, remains well below −20 dB whereas that between Ports 2 and 4 as well as Ports 1 and 3, is now improved to be consistently below −15 dB.

### 3.2. Measurement Validation

**Figure 14:**
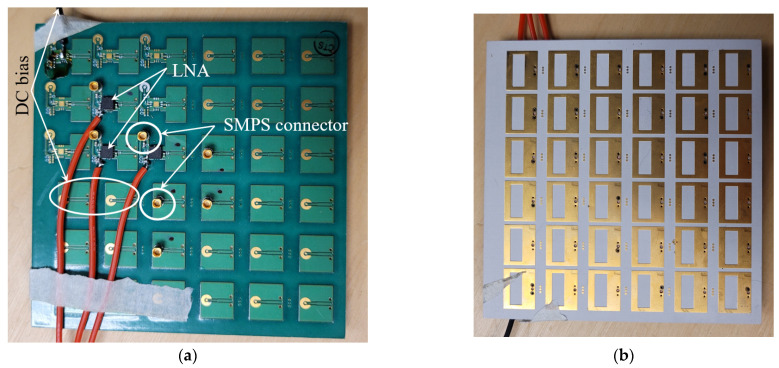
Photo of the original SMPS-connectorized 6 × 6 array described in [25]: (**a**) electronic layer, where 3 array elements are active (with LNA chips), (**b**) slot layer that comes in direct contact with tissue.

**Figure 15:**
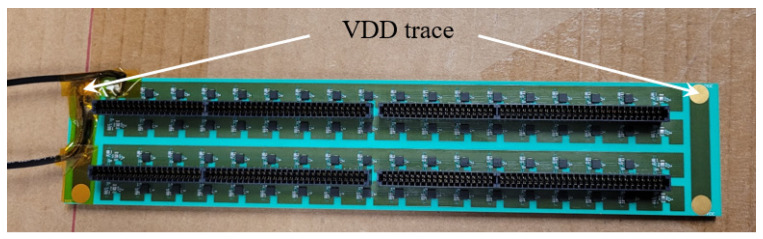
The electronics (top) layer of one of the five fabricated array tiles used to build a 16 × 16 UWB sensing array prototype. The slot (bottom) layer (not shown) contains 4 rows of 16 slots, which are identical to those in Figure 14b. The tile accommodates 64 active sensors and 8 dummy array elements at the left and right ends of the PCB. The active sensors are equipped with LNA chips whose outputs lead to the vertical connector pins.

**Figure 16:**
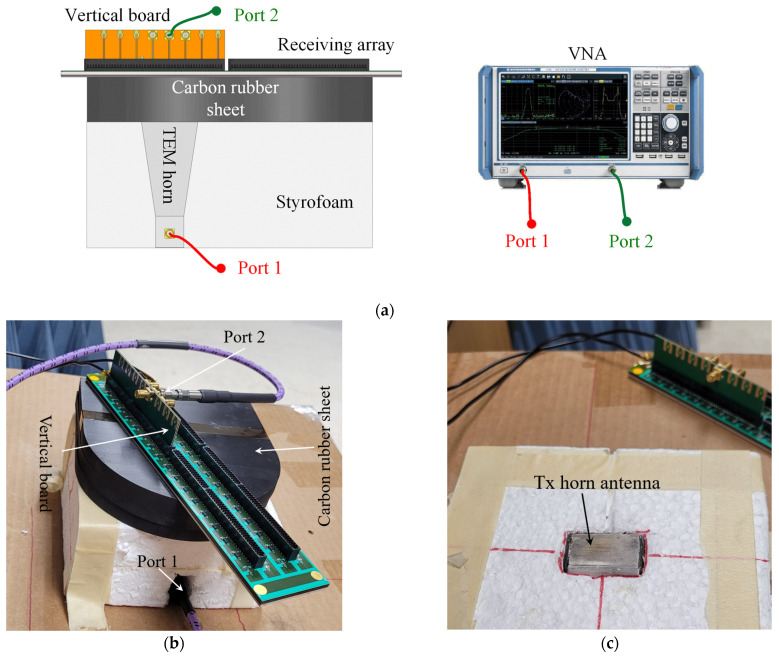
(**a**) The schematic of measurement setup of transmit-receive configuration. (**b**) The measurement setup of the transmit-receive configuration, where the Tx antenna and the Rx array assembly are separated by three 11 mm-thick circular carbon-rubber sheets, mimicking the electrical properties of healthy breast tissue. (**c**) TEM dielectric horn [37] used as the Tx antenna.

To compare the RF connectivity offered by the high-speed vertical connectors with that of the SMPS connectors, two prototypes of the UWB active sensing array are utilized. The first prototype is the original SMPS-connectorized 6 × 6 array described in [25]. Its photo is shown in Figure 14. The second prototype is the new 16 × 16 array (see Figure 2) designed to accommodate the vertical connector. As seen in Figure 2, a 16 × 16 array can be built from identical array tiles, each housing 64 active antenna elements. A photo of one such tile is shown in Figure 15. This tile is used to carry out the comparison with the original SMPS-connectorized 6 × 6 array in Figure 14. A vertical test board is also fabricated in accordance with the GCPW vertical-board design presented in Section 3.1. It is inserted in one of the vertical connectors of the Rx array tile as shown in the photo in Figure 16a.

As shown in Figure 16a, a transmit-receive setup is put together, where a dielectric-filled transverse electromagnetic (TEM) horn [37] is used as the Tx antenna; see Figure 16c. The Tx antenna and the Rx active array (either the SMPS-connectorized 6 × 6 array or the new array tile with the vertical connector) are placed on the opposite sides of a stack of three 11 mm-thick custom-made carbon-rubber slabs with complex permittivity of *ε*_r_ = 9.6 − i3.8 (averaged over the frequency band from 3 GHz to 8 GHz). The slab material mimics the dielectric properties of healthy breast tissue. Figure 16a shows the setup, where the Tx antenna is beneath the breast-tissue phantom. The Tx antenna is connected to Port 1 of the VNA, and its aperture comes in direct contact with the tissue phantom. On the opposite (top) side, the Rx array slots also come in direct contact with the breast phantom. One of these slots is aligned along the boresight with the TX horn antenna. This slot belongs to the active sensor element whose output on the vertical test board is equipped with an SMA connector, which in turn is connected to Port 2 of the VNA. The exact same Tx antenna, arrangement, and alignment are used when measuring the received signal with one of the elements of the SMPS-connectorized 6 × 6 array. We emphasize that the sensing elements of the 6 × 6 array and the new Rx array tiles are identical, except for the different connectors at their outputs.

**Figure 17:**
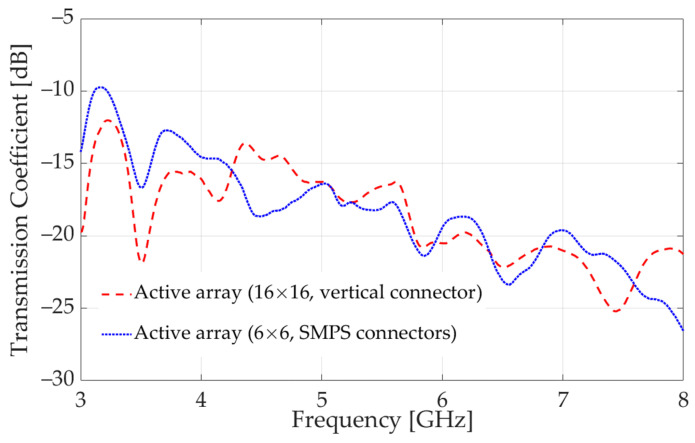
Measured “through” transmission coefficient with the two active Rx arrays: one employing a high-speed vertical connector and the other employing SMPS connectors.

The *S*_21_ (through) transmission coefficients (in dB, through the tissue phantom) measured with the two Rx arrays are shown in Figure 17. It is evident that the RF connection provided by the high-speed vertical connector is of comparable quality to that of the SMPS connectors. We note that the active Rx antenna elements on the PCB, including the vertical connectors and their interconnects, are expected to have identical reception performance. However, quantifying the array reception uniformity requires an equally repeatable transmission performance, which can be achieved only with precision alignment of the Tx and Rx antenna elements. The development of a printed Tx antenna array, which provides such alignment, is still ongoing.

## 4. Discussion

This study is an integral part of the development of a large-scale, densely packed electronically switched antenna array for biomedical imaging in the frequency range from 3 GHz to 8 GHz, with a focus on the receiving array. The IF-switched architecture enables the multiplexing of hundreds of array elements in contrast to the conventional RF-switched architecture, which is limited to several tens of sensors. While our IF-switched imaging system is a work in progress, preliminary studies suggest that employing switching circuits at the IF frequency of 30 MHz offers the following advantages over switching at RF frequencies in the UWB range: (i) insertion loss less than 1 dB compared to 6 dB at UWB, (ii) isolation better than 60 dB compared to 20 dB at UWB, (iii) return loss better than 25 dB compared to 10 dB in UWB, and (iv) easy design of the interconnecting transmission lines with superior amplitude and phase uniformity across all 256 channels.

However, an IF-switched sensing array requires the integration of each antenna with an RF front end. The limited space on the antenna board (12 mm in both *x* and *y* directions), coupled with the lack of a single-chip platform for low-noise amplification and frequency conversion over the wide frequency band (3 GHz to 8 GHz), dictates that the antenna array board can only accommodate the LNA chips. Therefore, a separate board becomes necessary to house the mixer array, the LO distribution network, and the IF-switching network. The seamless transition between these two boards is of paramount importance.

In this manuscript, the primary focus is on the development of mechanically robust, low-cost, and low-crosstalk connections between the UWB active antenna-array board and its corresponding mixer boards. SMPS connectors, due to size, cost, and reliability concerns, were found inadequate for the task. As an alternative, we turn to high-speed digital board-to-board vertical connectors, known for their high pin density, mechanical and electrical durability, and affordability. We explore a particular off-the-shelf connector (MEC2-50-01-L-DV) within the UWB frequency range for establishing efficient board-to-board microwave connections. The interfacing of this connector with microstrip lines is well-documented by the manufacturer. However, GCPW TLs are preferred in UWB technology. This study shows that, while the connector can indeed be connected to GCPWs on both the horizontal and vertical boards, it is not sufficient to simply attach a 50-Ω GCPW to the connector’s pins. Careful design and simulation-based optimization have been reported here that target not only the GCPW signal-trace and gap widths but also slots in the GCPW ground-plane layer as well as ground-plane de-metallization (partial ground plane). The proposed design achieves very good microwave performance with reflection loss better than 10 dB, insertion loss well below 1 dB, and mutual coupling (crosstalk) below −20 dB.

The designed GCPW interconnects for the high-speed vertical connector have been employed here to integrate a 16 × 16 UWB active sensing array (horizontal) board with vertical boards. However, the proposed board-to-board GCPW transitions can also be employed in many other UWB applications where simultaneous seamless microwave signal transfer is needed for hundreds of channels. 

It is worth noting that there is a great variety of other printed transmission lines, e.g., CPWs without a ground plane, differential, strip, and slot lines, and the ways to interface these with high-speed board-to-board connectors are yet to be investigated.

## 5. Conclusions

We have proposed an efficient method to interconnect hundreds of grounded-coplanar-waveguide (GCPW) input/output signal paths on two separate PCBs (one horizontal and the other vertical) based on widely available low-cost high-speed digital vertical connectors. This development enables the realization of large-scale high-density electronically scanned or switched antenna arrays, which are critical in wireless communications as well as in microwave radar, imaging, and sensing. Excellent RF performance has been demonstrated in the UWB band through simulations and measurements in terms of reflection loss, insertion loss, and channel crosstalk. 

## Data Availability

Not applicable.
